# Editorial: Genetics Architecture and Underlying Molecular Mechanisms in Host-Pathogen Interactions

**DOI:** 10.3389/fgene.2021.695109

**Published:** 2021-08-18

**Authors:** Benjamin L. Makepeace, Androniki Psifidi, Diego Robledo, Dong Xia

**Affiliations:** ^1^Department of Infection Biology and Microbiomes, Institute of Infection, Veterinary and Ecological Sciences, University of Liverpool, Liverpool, United Kingdom; ^2^Department of Clinical Science and Services, Royal Veterinary College, University of London, London, United Kingdom; ^3^The Roslin Institute and Royal (Dick) School of Veterinary Studies, University of Edinburgh, Edinburgh, United Kingdom; ^4^Department of Comparative Biomedical Science, Royal Veterinary College, University of London, London, United Kingdom

**Keywords:** host-pathogen interactions, “omics” and systems biology, genetics, epigenetics, one health

The study of host-pathogen interactions has been accelerating rapidly in both medical and veterinary contexts due to methodological advances and increasing infectious disease threats. This Research Topic attracted a wide breadth of submissions spanning analyses of host-pathogen interactions using *in vitro* and *in vivo* models of human disease; through to a range of studies in livestock species, comprising cattle, pigs, chickens, and farmed fish. An impressive breadth of technical approaches was also in evidence, from GWAS, SNP analysis, transcriptomics, RNAi, proteomics, and reverse genetics; often deployed in combination in a single study. Overall, there was as a strong bias toward “host-centric” articles (*n* = 12) compared with “pathogen-centric” (*n* = 6) studies, perhaps reflecting the worldwide drive to improve disease resistance in livestock.

Three “host-centric” studies were concerned with pathogens that are primarily of medical importance. Tan and Xia studied herpes simplex virus-1 (HSV-1) infection of the eye, a leading cause of infectious blindness, using a mouse model. Tripartite motif-containing protein-21 (TRIM21) was identified as a key factor in HSV epithelial keratitis. Upregulation of this host protein enhances the replication of HSV-1 in corneal epithelial cells *via* inhibition of stimulator of interferon genes/interferon regulatory factor-3 signalling and subsequent suppression of type I interferon production. TRIM21 also promotes the secretion of interleukin-6 and tumour necrosis factor-α, thereby aggravating the severity of HSV epithelial keratitis. Switching to bacterial pathogens, Wang et al. studied *Staphylococcus aureus* osteomyelitis in mice using both *in vivo* and *in vitro* systems. Based on an analysis of publicly available microarray data, they demonstrated the essential role of twist-related protein 1 (TWIST1) and NF-κB signalling in the early response to *S. aureus* infection in bone. The authors used a range of molecular techniques, including siRNA-mediated suppression of TWIST1, which impaired the migration and phagocytotic ability of macrophages in response to *S. aureus*. The third study on a human pathogen was that of Hsieh et al., who studied the role of intracellular NAD^+^ content in the response to Group A streptococcus in a human microvascular endothelial cell line-1 (HMEC-1). The authors discovered that treatment with nicotinamide increases intracellular NAD^+^ content, which enhances autophagy-mediated clearance of group A streptococcus in these cells. In contrast, nicotinamide did not have any impact on the infection by other intracellular bacteria.

Among the livestock host studies, two were focused on disease resistance in pigs. Sanglard et al. investigated the genomic architecture underlying the antibody response to porcine reproductive and respiratory syndrome virus (PRRSV) vaccination and its relationship to reproductive performance in commercial pigs. The trait was moderately heritable (h^2^ = 0.34) and one major locus was identified on chromosome 7 (in the major histocompatibility region, MHC). The antibody response was highly correlated with several reproductive traits, suggesting it has great potential as an indicator to improve reproductive performance in commercial pigs. In the other study, Bai X. et al. studied phenotypes related to disease resilience using complete blood count (CBC) data from a wean-to-finish natural disease challenge model in F1 crossbred (Landrace × Yorkshire) pigs. Resilient animals were found to be primed to initiate a faster adaptive immune response and recovered earlier following infection, with greater increases in lymphocyte concentration, haemoglobin concentration, and haematocrit values, but exhibited a lower neutrophil concentration than in susceptible animals, including those that died. The CBC traits were found to be heritable and genetically correlated with growth and treatment, which may indicate that this phenotype can be used as part of developing predictions for disease resilience.

Chickens were represented by three host-focused studies. Deist et al. explored the genomic factors that lead to increased resistance to Newcastle disease virus (NDV). The authors studied the transcriptome of three different tissues upon challenge with the virus in two chicken genetic lines showing differential resistance to NDV. The study revealed time- and line-dependent responses to the virus and the authors used a network correlation analysis to integrate all the results and identify key factors driving resistance, such as eukaryotic translation initiation factor 2-alpha kinase-2, macrophage-expressed gene 1 protein, and tumour necrosis factor ligand superfamily member 13B. Meanwhile, Liu R. et al. used transcriptomics and proteomics to study the response of chicken synovial fibroblasts to *Mycoplasma synoviae*, an important poultry pathogen that can cause respiratory disease and arthritis. The authors used a primary cell culture of synovial tissues from pathogen-free chickens. While transcriptomic and proteomic data showed relatively little overlap, the authors identified potentially important players in *M. synoviae*-induced arthritis, including proliferation and apoptosis related factors, inflammatory mediators, or proangiogenic factors. A different approach was taken by Banos et al., who performed a joint analysis of two distinct Ethiopian indigenous chicken ecotypes to investigate the genomic architecture of important health and productivity traits and explore the feasibility of conducting genomic selection across ecotypes. A GWAS identified several significant genomic associations with health and productivity traits, while whole genome sequencing and pathway analysis revealed putative candidate genes and mutations. Reliability of genomic estimated breeding values across ecotype increased compared to within-ecotype calculations, but accuracy of genomic prediction did not, probably because the genetic distance between the two ecotypes offset the benefit from increased sample size.

The increasing importance of aquaculture for protein production was reflected in three articles on the challenges of infectious diseases in farmed fish. Fraslin et al. presented a review on different methods used in fish research to dissect host-pathogen interactions. Identification of QTL underlying resistance to pathogenic microbes, transcriptomics studies, and functional assays that describe host–pathogen interactions are discussed. The utility of fish isogenic lines as well as the need to combine multiple approaches to better understand host-pathogen dynamics is also highlighted. In a primary research article, Mastrochirico-Filho et al. generated a *de novo* transcriptome for a neotropical fish, the small-scaled pacu (*Piaractus mesopotamicus*), which is of major importance for South American aquaculture. The authors use this transcriptome to study the immune responses of pacu to *Aeromonas hydrophila* during the acute mortality phase of the infection and after it decreases and plateaus. Suppression of genes of the complement system and coagulation factor cascades may contribute to the pathogenesis of *A. hydrophila*. Moving to the northern hemisphere, Król et al. explored the molecular mechanisms associated with the progression of multifactorial gill disease in Atlantic salmon. Gills from three different aquaculture sites in Scotland were sampled and examined *via* gross morphology, RNA sequencing and histopathology. Gene expression or histopathology did not directly correlate with the changes in morphology (gill damage). Nonetheless, the authors did identify common expression patterns associated with multifactorial gill disease, mainly connected to two processes: immune responses driven by pro-inflammatory cytokines, and tissue damage and repair driven by caspases and angiogenin.

The final livestock host study was that of Holder et al., who investigated the presence of SNPs in the bovine *SLC11A1* gene and any resulting functional differences in natural resistance-associated macrophage protein 1 expression (NRAMP1; controlled by *SLC11A1*) that might be correlated with resistance/susceptibility to *Mycobacterium bovis* infection. One such SNP was identified and further analysis using ELISA revealed that the presence of an alternative G allele was associated with increased expression of NRAMP1 in bovine macrophages. Since NRAMP1 has been shown to influence the survival of *M. bovis* through the sequestering of iron, it is possible that cattle expressing the alternative G allele might have an increased resistance to infection.

Despite their small number, the “pathogen-centric” studies were diverse in subject matter, including representatives of bacterial, protozoal, and helminth pathogens and an arthropod ectoparasite. Cui et al. performed a genomic analysis of 31 *Cronobacter* (Enterobacteriaceae) isolates representing multiple species to identify putative virulence factors. Genome sequences of these species were compared with virulence genes in a virulence factor database, and those identified exhibited species specificities. Two novel gene clusters associated with higher cytotoxicity and stronger haemolysis capacity were identified. Another Gramme-negative pathogen featured in the study of Park et al., who demonstrated the function of a polycistronic mRNA leader in *Salmonella* that secures the growth of the bacteria. The *mgtCBR* mRNA encodes the virulence protein MgtC and the Mg^2+^ transporter MgtB, while a mutant with leaderless *mgtCBR* mRNA dysregulated the expression of MgtC and MgtB, reducing ATP to abnormal levels in a process that did not require F_1_F_0_ ATP synthase. Growth of the mutant was shown to be impaired in both extracellular and intracellular (macrophage) environments. In another *in vitro* study, Peirasmaki et al. investigated the transcriptome of a protozoan, *Giardia intestinalis*, during its interaction with the human intestinal epithelial cell line, Caco-2. They identified members of the high cysteine membrane protein (HCMP) family to be among the most abundant upregulated genes. The authors subsequently located these HCMPs to the plasma membrane and peripheral vesicles and demonstrated that the expression level of these genes was affected by histone acetylation and the level of iron in the growth medium, linking them to potentially important roles during *Giardia*-host cell interactions.

The studies on multicellular parasites included Shi et al.'s proteomic characterisation of excretory-secretory products (ESP) and tegument proteins in the foodborne zoonotic liver fluke, *Clonorchis sinensis*. Enolase and cathepsin C were among the proteins identified in the ESP fraction, whereas annexins, actin, and tetraspanins were identified in tegument fraction as potential immunomodulators and promising vaccine antigens. A subset of tegument proteins labelled with biotin was localised to the surface membrane of the tegument. Different approaches were applied by Bai Y. et al., who employed RNA-seq and miRNA-seq to characterise the bi-directional development of protoscoleces in the dog tapeworm, *Echinococcus granulosus*. Comparisons between profiles of larval cysts and strobilar adult worms revealed that bi-directional development is controlled by miRNAs and genes likely associated with nervous system development and carbohydrate metabolic process. This information provides avenues to develop novel interventions and therapeutics for controlling the important zoonotic disease, cystic echinococcosis. Finally, continuing with the theme of small RNAs, Liu W-G. et al. characterised a novel tick-specific miRNA, hlo-miR-2, in *Haemaphysalis longicornis* that regulates moulting events; a process that is essential for the lifecycle of this three-host tick. The hlo-miR-2 and its predicted target gene *CPR1*, encoding a cuticular protein, exhibited tissue and temporal specificity; whereas overexpression or depletion of hlo-miR-2 led to a delayed or accelerated moulting process through the corresponding changes to *CPR1* expression.

In conclusion, this Research Topic attracted a broad range of host- and pathogen-centric studies employing a wide variety of methodological approaches. It highlights how sequencing technologies are rapidly “levelling the playing field” with respect to high-throughput investigations of host-pathogen interactions in livestock species, while previously these were largely the preserve of studies on humans or model organisms. Combined with continues improvements in both host and pathogen genome annotations and assemblies, as well as recently developed domain and expression based host-pathogen protein interaction predictions (Kshirsagar et al., 2013) and agent-based and dynamic optimization at system level (Pollmacher and Thilo Figge, 2015; Balsa-Canto et al., 2016), a genomic view of host-pathogen interaction models is emerging. A major theme was improving our understanding of disease resistance in livestock and its heritability, but genetic mechanisms underpinning phenotypic regulation in pathogens was also the topic of several papers. As we enter a new era with a belated appreciation of infectious disease threats, the study of host-pathogen interactions and disease susceptibility in wildlife, domestic animals and humans should take on a new urgency.

## Author Contributions

All authors contributed to this Editorial and co-edited the corresponding Research Topic.

## Conflict of Interest

The authors declare that the research was conducted in the absence of any commercial or financial relationships that could be construed as a potential conflict of interest.

## Publisher's Note

All claims expressed in this article are solely those of the authors and do not necessarily represent those of their affiliated organizations, or those of the publisher, the editors and the reviewers. Any product that may be evaluated in this article, or claim that may be made by its manufacturer, is not guaranteed or endorsed by the publisher.
